# Self-Care Index and Post-Acute Care Discharge Score to Predict Discharge Destination of Adult Medical Inpatients: Protocol for a Multicenter Validation Study

**DOI:** 10.2196/21447

**Published:** 2021-01-14

**Authors:** Antoinette Conca, Daniel Koch, Katharina Regez, Alexander Kutz, Ciril Bächli, Sebastian Haubitz, Philipp Schuetz, Beat Mueller, Rebecca Spirig, Heidi Petry

**Affiliations:** 1 Department of Nursing Science University Witten/Herdecke Witten Germany; 2 Medical University Department, Division of General Internal and Emergency Medicine Kantonsspital Aarau Aarau Switzerland; 3 Centre of Clinical Nursing Research and Development University Hospital Zurich Zurich Switzerland

**Keywords:** discharge planning, forecasting, logistic models, patient transfer, post-acute care discharge score, protocol, self-care index, sensitivity, specificity, validation study

## Abstract

**Background:**

Delays in patient discharge can not only lead to deterioration, especially among geriatric patients, but also incorporate unnecessary resources at the hospital level. Many of these delays and their negative impact may be preventable by early focused screening to identify patients at risk for transfer to a post-acute care facility. Early interprofessional discharge planning is crucial in order to fit the appropriate individual discharge destination. While prediction of discharge to a post-acute care facility using post-acute care discharge score, the self-care index, and a combination of both has been shown in a single-center pilot study, an external validation is still missing.

**Objective:**

This paper outlines the study protocol and methodology currently being used to replicate the previous pilot findings and determine whether the post-acute care discharge score, the self-care index, or the combination of both can reliably identify patients requiring transfer to post-acute care facilities.

**Methods:**

This study will use prospective data involving all phases of the quasi-experimental study “In-HospiTOOL” conducted at 7 Swiss hospitals in urban and rural areas. During an 18-month period, consecutive adult medical patients admitted to the hospitals through the emergency department will be included. We aim to include 6000 patients based on sample size calculation. These data will enable a prospective external validation of the prediction instruments.

**Results:**

We expect to gain more insight into the predictive capability of the above-mentioned prediction instruments. This approach will allow us to get important information about the generalizability of the three different models. The study was approved by the institutional review board on November 21, 2016, and funded in May 2020. Expected results are planned to be published in spring 2021.

**Conclusions:**

This study will provide evidence on prognostic properties, comparative performance, reliability of scoring, and suitability of the instruments for the screening purpose in order to be able to recommend application in clinical practice.

**International Registered Report Identifier (IRRID):**

DERR1-10.2196/21447

## Introduction

Delays in hospital discharge are associated with deterioration in the performance of activities of daily living (ADL), especially among frail patients [1,2], and other negative patient outcomes like hospital-acquired infection (pneumonia, urinary tract infection, or sepsis), short- and long-term mortality [3], and negative economic outcomes like hospital costs [4].

In frail elderly patients, nonmedical reasons accounted for nearly one-third of prolonged hospitalization, with nursing facility placement delay [5] and waiting time for post-acute care (PAC) institutions being the most common reasons [4,6,7]. Delayed hospital discharge is therefore caused to a relevant extent by non-medical reasons (288/960, 30% [7]; 392/1221, 32.1% [8]), such as no free beds being available in a nursing home or PAC facility, discharge to home not being possible, or delivery of nursing/medical equipment at home being delayed) [7-9]. In hospitalized patients with respiratory tract infections, organizational issues caused delayed discharge [10], even with structured discharge planning [11]. Furthermore, prolonged hospital stays due to nursing and organizational reasons were evident in patients with decompensated heart failure [12].

Several studies concur on a list of risk factors of discharge to follow-up care institutions: advanced age, living alone, functional disability, and preexisting ADL and instrumental ADL limitations [13-15].

The fact that so many discharges of medically stable patients are delayed due to lack of resources in PAC institutions indicates a need to refine the processes of both triage and early interprofessional discharge planning [6,10-12,16-18].

Two instruments have been developed to identify patients requiring transfer to PAC facilities (the post-acute care discharge score [PACD]; see Multimedia Appendices 1 and 2) or to predict postdischarge care needs (the self-care index [SPI]; see Multimedia Appendix 3). The PACD scores were developed to identify medical patients requiring transfer to a PAC facility [6]. Patients who score less than 8 points on the PACD are considered at low risk for requiring post-acute care. A score of 8-15 points defines medium risk, and more than 15 points indicates a high risk for requiring post-acute care [6]. Two versions, PACD day-1 (15 items) and day-3 (5 items), were published [6]. Both showed good performance (day-1 area under the curve (AUC)=0.81; day-3 AUC=0.82). Our team further developed the score, and in patients with respiratory tract infections (n=240), biopsychosocial risk (PACD day-1) correlated significantly with discharge to a PAC facility [19]. Both versions showed an acceptable sensitivity and specificity (cutoff at ≥8, PACD day-1: sensitivity 82%, specificity 55%, AUC=0.90; PACD day-3: sensitivity 86%, specificity 69%, AUC=0.79) [20]. In patients admitted from home with urinary tract infections, falls/syncope, or heart failure (n=308), PACD day-1 showed a sensitivity of 90% and a specificity of 62%, and PACD day-3 showed a sensitivity of 80% and a specificity of 60%, with cutoff at ≥8 [21]. The accuracy was good for both the day-1 (AUC=0.82) and the day-3 (AUC=0.79) versions [21]. Validated in a prospective cohort study with 1432 medical and 464 neurological patients, PACD day-1 and day-3 provided AUCs of 0.77 and 0.82, sensitivities of 72.6% and 83.6%, and specificities of 66.5% and 70.0% with cutoff at ≥8. Neurological patients’ scores showed lower accuracy both days: AUCs were 0.68 and 0.78, sensitivities were 41.4% and 68.7%, and specificities were 81.4% and 83.4% [22].

The SPI was developed to identify a possible care deficit after hospitalization (former name: “CaseManagementScore,” CMS) and is widely used as a patient assessment instrument in hospitals across German-speaking regions. Patients who scored more than 32 points on the SPI are considered at low risk for having a care deficit after a hospital stay. A score of less than or equal to 32 points on the SPI indicates a high risk for having a care deficit. In a consecutive sample of 620 hospital patients (cutoff≤32), the SPI yielded 85.5% sensitivity and 92.3% specificity [23].

In a cohort of 1342 medical and 402 neurological patients, both PACD and SPI predicted transfer to PAC facilities (*P*<.001). SPI sensitivity was 64% and the specificity was 84%, with cutoff≤32. The PACD combined with SPI (AUC=0.83, +7.8%; 0.78, +14.7%) identified patients at risk significantly better than the PACD alone (AUC=0.77; 0.68). Also, sensitivities (68%, −6.8%; 55%, +37.5% vs. 73%; 40%) and specificities (82%, +26.2%; 85%, +4.9% vs. 65%; 81%) were mostly higher than with the PACD alone. Patients who scored less than 16 points on the PACD-SPI combination are considered at low risk for requiring post-acute care. A score of 16-25 points was considered as medium risk, and more than 25 points is considered a high risk for requiring post-acute care. The net reclassification index (28.6%) and integrated discrimination index (4.8%) both showed significant (*P*<.001) improvement [24]. The results indicate that self-care abilities are an independent predictor for the risk of PAC facility discharge [24].

In the described preceding studies, PACD scores accurately predicted transfer to PAC facilities, indicating potential as screening instruments to improve discharge planning and shorten hospital length of stay. A review on prediction of support services after hospital confirmed the particular need for external validation studies [25]. As the results were replicated in a monocenter study only, the predictive accuracy requires further validation in other hospitals and regions in a multicenter setting with a larger and more varied sample of medical patients.

The research proposed in this study protocol aims to explore the ability of SPI to predict post-acute care discharge, develop scoring, and then test whether PACD (day-1), SPI, or the combination of PACD and SPI can reliably identify patients requiring transfer to PAC facilities in order to replicate previous findings and allow generalization.

## Methods

### Overview

We follow the recommended procedures by the Prognosis Research Strategy (PROGRESS) Group [26-28] and report according to the TRIPOD (Transparent Reporting of a Multivariable Prediction Model for Individual Prognosis or Diagnosis) statement [29,30]. The PROGRESS guidelines on predictive modelling [28] advise testing of scores in new settings. In our research we apply the scores in different hospital types (urban, rural) and other geographical areas. Therefore, the proposed multicenter external validation results are needed to test the calibration and, depending on the results, suggest a recalibration of the scores.

### Study Design

This study is embedded in a 3-phase pre-post quasi-experimental study called “Integrative hospital treatment in older patients to benchmark and improve outcome and length of stay – the In-HospiTOOL study,” conducted at secondary and tertiary hospitals in both urban and more rural areas in the German-speaking part of Switzerland, aiming to safely reduce hospital length of stay by implementing an interprofessional discharge management tool [31,32].

### Study Population (Sample and Setting)

We will include consecutive, unselected adult medical patients admitted to the hospitals through the emergency department within the In-HospiTOOL study, using both the PACD and SPI instruments for patient assessment (4 hospitals). We will exclude patients admitted from PAC facilities (eg, nursing homes), patients transferred from or to another hospital, and patients who die during the study period. We estimate that we will include a patient sample of 3000 in the observation period (6 months) to check the scoring for the SPI and 6000 in the subsequent period (12 months) with both the PACD and SPI instruments during the data collection phase.

### Data Collection

Data will be collected by health professionals from July 1, 2017, to February 8, 2019, from eligible medical patients admitted to hospital during the study period as part of routine clinical care documentation. Health professionals will be trained by an instruction video and one-hour on-site workshop. Data will then be exported from the clinical information system to the study database. The physicians who initially assess the patients will indicate the number of active medical problems at admission, defined as International Classification of Diseases diagnoses with diagnostic or therapeutic consequence for actual treatment, increased monitoring needs, or both. The treating physicians and nurses will assess the PACD day-1 scores in the emergency department or on the ward, depending on their hospital’s processes within 24-48 hours. The SPI will be collected by the staff nurse as part of the standard nursing assessment (“ergebnisorientiertes Patientenassessment–Acute Care” [ePA-AC]) within the first 2 days of admission. The medical coding department will provide data on pre-admission and postdischarge residence and length of stay from Diagnosis Related Group coding data collected for the Swiss Federal Statistical Office.

### Predictors

#### PACD

The PACD includes information on the patient’s age, active medical problems, and support situation at home within the last 14 days, while integrating his or her abilities in ADL and instrumental ADL [21] (see Multimedia Appendix 1). The PACD was translated from French to German by the research team [19] and pilot-tested regarding comprehensibility and clinical practicability on 10 medical patients in the emergency department setting. After the test, we adapted the PACD’s phrasing accordingly [19].

In the original PACD day-1, no scoring was defined, because only the PACD day-3 was implemented at the study site in Geneva, Switzerland. Therefore, for the PACD day-1’s first tests, conducted at the Aarau Cantonal Hospital (a 600-bed teaching hospital in Switzerland), the principles for point definition used by the authors for the scoring of the day-3 version [6] were applied [19,21]. After comparing the predictors in the logistic regression model, we allocated points depending on how much larger or smaller the other standardized regression coefficients were [19]. Proportional points per answer were defined based on their value in relation to each other. The one exception, based on clinical considerations, was the decision to allocate 1 risk point for each 10 years of age, starting at 60 (1 point), with a maximum of 5 points for patients 100 years or older (see Multimedia Appendix 1) [19].

Based on this analysis, two adaptations were made [22]. First, “transfer within the hospital” (part of the original PACD day-1) [6] was omitted because it was not significantly predictive of PAC facility transfer [22]. Second, “partner to provide help” was modified to “someone living with the patient to provide help” [10,19,22].

#### SPI

The SPI assesses the degree of patients’ self-care, and Grosse Schlarmann [23] examined this part of the “Result-Oriented Nursing Assessment–Acute Care” (52-item ePA-AC version 1.0) as a screening tool to identify postdischarge nursing care deficits.

The SPI includes 10 items with 4 Likert-type answer categories: mobility, personal hygiene (upper/lower body), dressing and undressing, eating and drinking, excretion of urine/ stool and cognition. The categories are completely dependent (1 point), requirement of extensive support (2 points), requirement of minor support (3 points), and independent (4 points), summing up to a total with a possible range of 10-40 points, where a score of 10 points corresponds to completely dependent. The SPI is usually measured as part of the standard nursing assessment within the first days after admission [19]. The cutoff point indicating a risk for PAC deficit was defined by the developer at less than or equal to 32 points [23].

### Outcome Measure

The primary outcome will be discharge destination, defined as transfer to a PAC facility (ie, temporary care, transient nursing care, health resort treatment, rehabilitation or nursing home) or discharge home. This information will be extracted from the discharge summary by the medical coding staff. They will be blinded to the scores.

### Power Calculation

To provide 60-100 degrees of freedom for our multivariable models, we aim to include a total of 6000 patients over the course of 12 months (both instruments implemented), with an expected 10% rate of PAC facility transfers (n=600). The expected rate is based on previous studies in Switzerland, where we found PAC transfer rates of 10.6% (152/1432) [22], 11.2% (150/1342) [24], and 16.7% (62/371) [21]. These percentages are lower than in 150 out of 752 patients (19.9%) discharged to facilities in an American hospital setting [33]. For all phases, the expected rate of outcome events (non-home discharge) exceeds the recommended 250 [34]. Power calculations for these models indicate that this sample size will have enough power to provide sufficiently precise confidence intervals regarding AUC, sensitivity, specificity, and positive and negative likelihood ratios, as well as for intergroup comparisons (power=80%) and logistic regression models.

### Data Handling

Data will be checked for patterns of missing responses and outliers. Patients with missing data will be compared based on discharge destination, age, active medical problems, and length of stay. Depending on the amount of missing data and their nonrandomness, they will be replaced by multiple imputation, or only complete cases will be included in the analysis.

### Data Availability

Data set will be made available on Dryad [35].

### Statistical Analysis

Patient characteristics will be analyzed descriptively using frequencies, percentages, medians, means, and standard deviations based on the data types and variable distributions, and we will compare the distribution of important variables (demographics, predictors, and outcome) between data sets (development and validation). The number of outcome events will be reported.

In order to validate the SPI for the purpose of screening and identifying patients likely to need institutional aftercare, the first step needed is to check the relation between SPI items and the transfer to a PAC facility and define optimal scoring of the SPI. This scoring combines the different self-care abilities with specific weights into a new risk score, which then needs to prove prognostic accuracy following a formative proceeding. For predictive scores, formative evaluation is recommended [36,37]. Therefore, we will derive a SPI model. To develop or refine the scoring if necessary, logistic regression models including the items of both scores as formative indicators will be analyzed, and the coefficients will be compared with existing data or examined for the need of weighting the items differently depending on their specific impact regarding transfer to a PAC facility.

Logistic regression models will be used to investigate the instruments’ individual and combined scores’ predictive capacities. We will present the full prediction model. The geographical external validation will take place in different hospitals in Switzerland.

We will use receiver operating characteristic analysis to estimate various cut-off points for sensitivity, specificity, positive predictive value, negative predictive value, and positive and negative likelihood ratios.

As the PACD and SPI are under consideration as screening tools for implementation in clinical practice, they would ideally identify every potential patient at risk of requiring PAC facility transfer and, therefore, comprehensive further assessment. While they do not achieve this standard, for their intended purpose, high sensitivity and adequate specificity are indicated.

Model calibration will be evaluated graphically and by test [30]. A calibration plot will be used to compare predicted transfer probability to observed transfer frequency. We will test for agreement between predicted and observed probabilities using the Hosmer-Lemeshow goodness-of-fit test [30,38]. To judge discrimination, we will calculate AUC (C statistic) with confidence intervals [39]. To test AUC differences between various scores, we will use a nonparametric approach including the “roccomp” procedure (Stata) [40].

We plan to assess the incremental prognostic value of SPI when added to the PACD score and therefore updating the prognostic model by the likelihood ratio test [30]. Classifications from the two models will be compared for changes by cross-table or scatterplot with smooth curve fitted by Loess [39]. The reclassification calibration will compare observed and predicted values within cross-classified categories [39]. Weighted net reclassification improvement [41] will be calculated. An integrated discrimination improvement analysis [42] will be performed and visualized by box plots [39].

To assess overall predictive performance in comparison, we will use the R^2^ Brier score and a validation graph [39]. We will judge overfitting by calibration slope [34,43].

Clinical usefulness will be judged by decision analytic measures. We will calculate the net benefit of using the models at a defined threshold (cross-table) with sensitivity analysis on different other thresholds [41], plot a decision curve [44], and determine the change in relative utility [41]. Decision curve analysis will provide evidence over a range of thresholds [39].

Values of *P*<.05 will be considered statistically significant. Reporting the 95% confidence intervals allows the reader to estimate the precision of the values [45]. Statistical analyses will be performed using SPSS Version 24.0 (IBM Corporation) and Stata Version 15.1 (StataCorp).

## Results

The study was approved by the institutional review board on November 21, 2016, and funded in May 2020. Expected results are planned to be published in spring 2021 (see Multimedia Appendix 4).

## Discussion

Evidence-based knowledge regarding implementation of an early assessment test is warranted to support clinical teams, accelerate discharge management, and determine the most appropriate post-acute care transfer destinations for patients at risk. The interprofessional PACD score and the SPI nursing score will serve as bases for discussion between health care professionals, with the potential to strengthen cultures of interprofessional teamwork. In addition to patient’s risk scores for PAC transfer, decision-making regarding discharge relies upon a mix of subjective clinical experience and objective data. If hospital stays can be shortened via a more process- and patient-oriented screening approach, the benefits will far outweigh the cost (in time) of assessing the scores.

We expect several potential limitations: as the PACD is newly implemented into routine care, and data collection depends on the completeness of documentation, there is a rate of missing data of at least 20%-30% anticipated. The PACD is combining the information of physician and nurses, which also increases the risk of gaps in information. Furthermore, to test the combination score, both measures need to be completed. The PACD, SPI, and combined (PACD/SPI) scores will be included in patient records as part of discharge planning by physicians, nurses, and social workers. All centers already worked routinely with the SPI; the PACD was newly introduced, and instructions were given for its use as basic information for discharge planning. Given this method of data collection, it is impossible to blind the study. Like other studies with similar population and sample size (n=885 to 1055) [46,47], the unselected and extensive patient data in other hospitals will provide a sufficient basis for robust analysis.

The instrument’s reliability and ability to reliably predict PAC facility transfer needs have to be tested in diverse populations to enhance the level of evidence, which is needed before safe recommendation and large-scale implementation. This external validation study will provide evidence on the incremental value of combining PACD and SPI for prediction of PAC transfer.

## Appendix

Multimedia Appendix 1 PACD day-1.

Multimedia Appendix 2 PACD day-3.

Multimedia Appendix 3 SPI Self-care index.

Multimedia Appendix 4 Project timeline.

## Abbreviations

ADL: activities of daily living
AUC: area under the curve
ePA-AC: ergebnisorientiertes Patientenassessment–Acute Care (Result-Oriented Nursing Assessment–Acute Care)
PAC: post-acute care
PACD: post-acute care discharge score
PROGRESS: Prognosis Research Strategy
SPI: self-care index
TRIPOD: Transparent Reporting of a Multivariable Prediction Model for Individual Prognosis or Diagnosis
