# Supplementary material for: Self-Care Index and Post-Acute Care Discharge Score to Predict Discharge Destination of Adult Medical Inpatients: Protocol for a Multicenter Validation Study
Source: JMIR Res Protoc. 2021 Jan 14;10(1):e21447. doi: 10.2196/21447 (PMC7843199; doi:10.2196/21447)
Supplement: Multimedia Appendix 1 [file resprot_v10i1e21447_app1.docx]

| Number of active medical problems on admission | | | | | |  | |
| --- | --- | --- | --- | --- | --- | --- | --- |
| Do you live with someone who can help you at home? | | | | | |  | |
| 🔾Yes 🔾No | | | | | | | |
| 🔾 Independent in all aspects at home | | | | | | |  |
| 🔾 Has needed help with … in the last 2 weeks: | | | | | | | |
| ❒ Personal hygiene | | ❒ Bathing / showering | ❒ Transfer bed/chair | ❒ Cooking | | | |
| ❒ Dressing / Undressing | | ❒ Eating / drinking | ❒ Car or public transportation | ❒ Housekeeping | | | |
| ❒ Toileting | | ❒ Moving | ❒ Shopping | ❒ Medication management | | | |
| Age: |  | | | | | | |
| **Total Score** | | | | |  | | |

Scoring of PACD day-1:

| Number of active medical problems: all current diagnoses of conditions with recognized therapeutic or diagnostic consequences are allocated one point for each affected organ system (Louis Simonet, personal communication on 17.05.2010). |
| --- |
| If the patient does not live with someone at home who can help, 4 points are allocated. |
| One additional point is added for each activity which could not be performed independently (activities of daily living and instrumental activities) during the last two weeks. |
| Age: 1 point is allocated for every 10 years of age starting at the age of 60, to a max. of 5 points for patients older than 99 years. |
