# Supplementary material for: Self-Care Index and Post-Acute Care Discharge Score to Predict Discharge Destination of Adult Medical Inpatients: Protocol for a Multicenter Validation Study
Source: JMIR Res Protoc. 2021 Jan 14;10(1):e21447. doi: 10.2196/21447 (PMC7843199; doi:10.2196/21447)
Supplement: Multimedia Appendix 2 [file resprot_v10i1e21447_app2.docx]

| Number of medically active problems on admission | |  | |
| --- | --- | --- | --- |
| Do you live with someone, who can help you at home? | |  | |
| 🔾 Yes 🔾 No | | | |
| Inability in medication self-management before admission | | |  |
| 🔾 Yes 🔾 No | | |  |
| Dependency for transfers bed/chair on the 3rd day. | | |  |
| 🔾 dependent 🔾 independent | | |  |
| Dependency for bathing/taking a shower on the 3rd day. | | |  |
| 🔾 dependent 🔾 independent | | |  |
| Total Score |  | | |

Scoring of PACD day-3:

| Active medical problems at admission (as w. PACD day-1, 1 point for each affected organ system) |
| --- |
| If the patient does not live with someone at home who can help, allocate 4 points (as w. day-1) |
| If the patient needed help with medication management before hospital admission, allocate 4 points |
| Dependency with transfer bed / chair on day 3: 4 points |
| Dependency with bathing / showering on day 3: 4 points |
