# Supplementary material for: Self-Care Index and Post-Acute Care Discharge Score to Predict Discharge Destination of Adult Medical Inpatients: Protocol for a Multicenter Validation Study
Source: JMIR Res Protoc. 2021 Jan 14;10(1):e21447. doi: 10.2196/21447 (PMC7843199; doi:10.2196/21447)
Supplement: Multimedia Appendix 3 [file resprot_v10i1e21447_app3.docx]

| 1 | self-care ability activity/movement |
| --- | --- |
| 2 | self-care ability in personal hygiene upper body |
| 3 | self-care ability in personal hygiene lower body |
| 4 | self-care ability in dressing and undressing upper body |
| 5 | self-care ability in dressing and undressing lower body |
| 6 | self-care ability ingestion: food |
| 7 | self-care ability ingestion: drink |
| 8 | self-care ability urine excretion |
| 9 | self-care ability stool excretion |
| 10 | ability to acquire knowledge |
|  | Levels of ability:  1 = "no ability ", 2 = “severely impaired ability”, 3 = “low impaired ability”, and 4 = “full ability” |
| Total | Sum of points of the Self-care Index (SPI)  ranging from 10 (completely dependent) to 40 (completely independent) |
