# Supplementary material for: Self-Care Index and Post-Acute Care Discharge Score to Predict Discharge Destination of Adult Medical Inpatients: Protocol for a Multicenter Validation Study
Source: JMIR Res Protoc. 2021 Jan 14;10(1):e21447. doi: 10.2196/21447 (PMC7843199; doi:10.2196/21447)
Supplement: Multimedia Appendix 4 [file resprot_v10i1e21447_app4.docx]

| **Objectives** | Pre-  period | Jul-Dec 2017 | Jan-Jul 2018 | Aug-Dec 2018 | Jan-Jun 2019 | Jul-Dec 2019 | Jan-Jun 2020 | Jul-Dec 2020 |
| --- | --- | --- | --- | --- | --- | --- | --- | --- |
|  |  |  |  |  |  |  |  |  |
| Ethical approval, investigator meetings (2x), planning & pilot phase |  |  |  |  |  |  |  |  |
| Preparation of subproject, exposé |  |  |  |  |  |  |  |  |
| Observational phase, active patient enrollment |  |  |  |  |  |  |  |  |
| Implementation phase, active patient enrollment main study |  |  |  |  |  |  |  |  |
| Intervention phase, active patient enrollment main study |  |  |  |  |  |  |  |  |
| Finishing follow-up, database finalization |  |  |  |  |  |  |  |  |
| Data analysis and manuscript preparation |  |  |  |  |  |  |  |  |
